# Network Pharmacology and Molecular Docking-Based Mechanism Study to Reveal the Protective Effect of Salvianolic Acid C in a Rat Model of Ischemic Stroke

**DOI:** 10.3389/fphar.2021.799448

**Published:** 2022-01-27

**Authors:** Yuting Yang, Yu He, Xiaoyu Wei, Haitong Wan, Zhishan Ding, Jiehong Yang, Huifen Zhou

**Affiliations:** Zhejiang Chinese Medical University, Hangzhou, China

**Keywords:** salvianolic acid C, ischemic stroke, network pharmacology, molecular docking, animal experiments

## Abstract

Salvianolic acid C (SAC) is a major bioactive component of *Salvia miltiorrhiza Bunge* (Danshen), a Chinese herb for treating ischemic stroke (IS). However, the mechanism by which SAC affects the IS has not yet been evaluated, thus a network pharmacology integrated molecular docking strategy was performed to systematically evaluate its pharmacological mechanisms, which were further validated in rats with cerebral ischemia. A total of 361 potential SAC-related targets were predicted by SwissTargetPrediction and PharmMapper, and a total of 443 IS-related targets were obtained from DisGeNET, DrugBank, OMIM, and Therapeutic Target database (TTD) databases. SAC-related targets were hit by the 60 targets associated with IS. By Gene ontology (GO) functional annotation and Kyoto Encyclopedia of Genes and Genomes (KEGG) pathway enrichment combined with the protein-protein interaction (PPI) network and cytoHubba plug-ins, nine related signaling pathways (proteoglycans in cancer, pathways in cancer, PI3K-Akt signaling pathway, Focal adhesion, etc.), and 20 hub genes were identified. Consequently, molecular docking indicated that SAC may interact with the nine targets (F2, MMP7, KDR, IGF1, REN, PPARG, PLG, ACE and MMP1). Four of the target proteins (VEGFR2, MMP1, PPARγ and IGF1) were verified using western blot. This study comprehensively analyzed pathways and targets related to the treatment of IS by SAC. The results of western blot also confirmed that the SAC against IS is mainly related to anti-inflammatory and angiogenesis, which provides a reference for us to find and explore the effective anti-IS drugs.

## Introduction

Stroke is a severe disease caused by cerebral blood circulation disorders, which can lead to high disability rate and high mortality, and has become one of the three most prevalent and serious diseases in the world (Krishnamurthi et al., 2020; Ajoolabady et al., 2021). Actually, stroke can be divided into ischemic stroke (IS) and hemorrhagic stroke, and up to 87% of stroke patients in the world are attributed to IS (Alexandru et al., 2017; Moon et al., 2021). The pathological process of IS is mainly manifested by focal cerebral ischemia, hypoxia and nerve necrosis (Jayaraj et al., 2019; Orellana-Urzúa et al., 2020). When IS occurs, due to the interruption of blood supply and the destruction of blood brain barrier (BBB), cells in the brain can’t maintain normal physiological functions. Then neuron damage and death may occur in the presence of energy depletion, oxidative stress, excitotoxicity, inflammatory response and other pathological factors (Yang et al., 2018; Zhou et al., 2021). The therapeutic effect of existing drugs is not ideal (Ciccone et al., 2013; Saver et al., 2015). Therefore, more effective drugs and measures are urgently needed to prevent and treat this disease.

**FIGURE 1:**
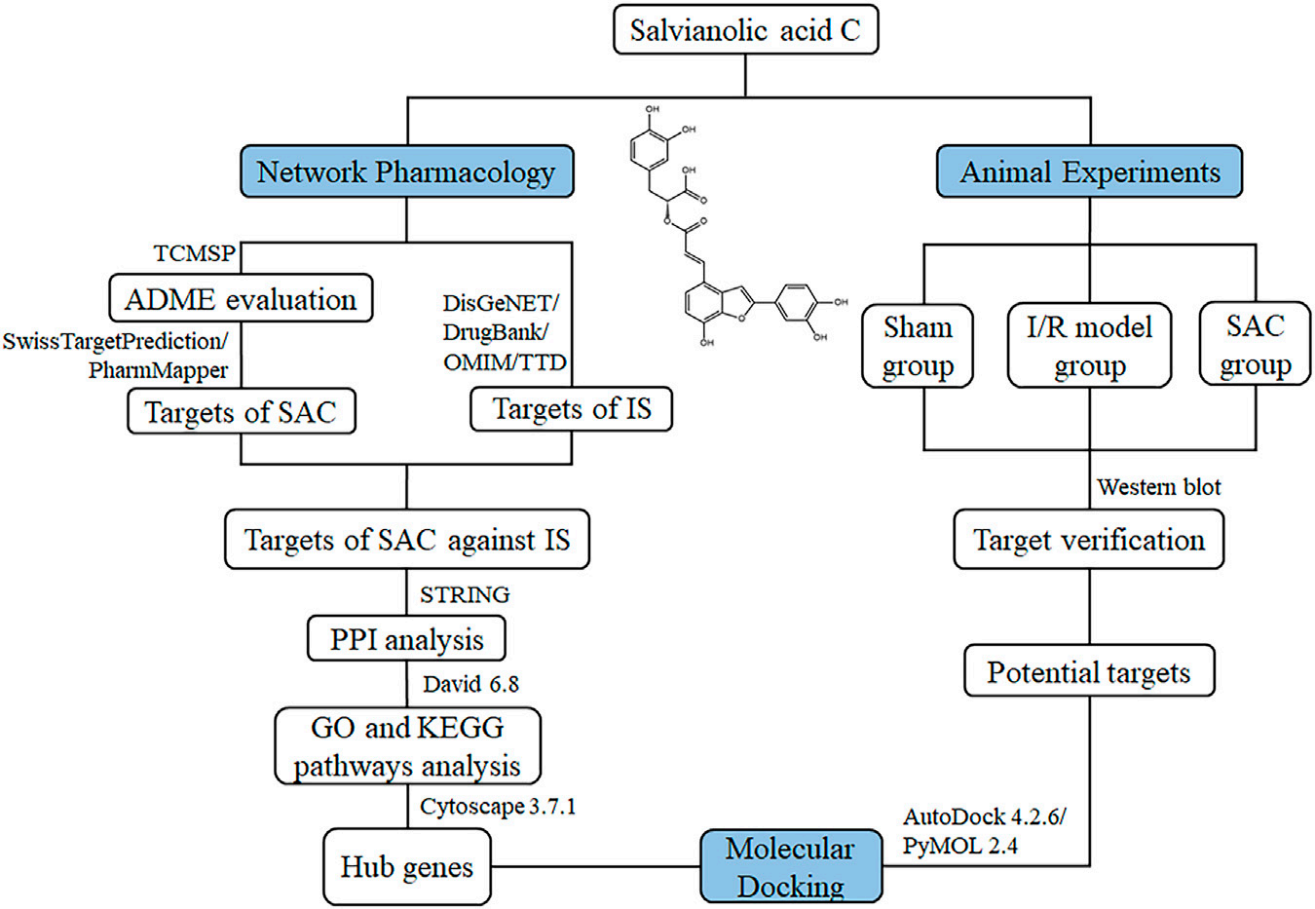
Chemical structure of SAC and flowchart of the mechanisms of SAC against IS.

Traditional Chinese medicine (TCM) has a long history of treating cardiovascular and cerebrovascular diseases and shows clinical efficacy (Gong and Sucher, 2002; Huang et al., 2015; Peng et al., 2019). *Salvia miltiorrhiza Bunge* (Danshen), a typical TCM, plays an important role in the compatibility of TCM and can be applied to treat IS (Zhou et al., 2005; Chen et al., 2013; Hung et al., 2015). Salvianolic acid C (SAC; Figure 1) is one of the water-soluble active components extracted from Danshen (Chen et al., 2016), with good drug-likeness. Currently, it has been reported that SAC has some pharmacological properties related to cerebral ischemia, including anti-inflammation, anti-oxidation, anti-platelet aggregation, and anti-apoptosis mechanisms (Song et al., 2018; Duan et al., 2019; He et al., 2019), but the functional mechanisms of SAC in the treatment of ischemic stroke is still unknown.

Network pharmacology is a cross-discipline that integrates the basic theories and research methods of network science, bioinformatics, computer science and mathematics, which analyzes the network of biological systems and selects specific signal nodes for drug molecular design (Hopkins, 2007; Song et al., 2019). As a novel holistic, a multi-disciplinary, integrative field, it views the drug-target interactions through the lens of systems-based approaches which provides a new framework for innovative drug discovery (Kibble et al., 2015; Song et al., 2019; Luo et al., 2020). And molecular docking is a method of drug design through the interaction between receptors and drug molecules (Dong et al., 2018; Pinzi and Rastelli, 2019). In recent years, it has been widely used in drug research and discovery (Ferreira et al., 2015; Pinzi and Rastelli, 2019). In this work, the potential action of SAC against ischemic stroke was predicted using network pharmacology. Additionally, molecular docking analysis and western blot assays were performed to confirm the mechanism of action of SAC on rat model of ischemic stroke. The procedures of this study were shown in Figure 1. SwissTargetPrediction and PharmMapper database were used to predict the SAC-related targets. DisGeNET, DrugBank, OMIM, and Therapeutic Target database (TTD) were used to search for targets of IS. After obtaining co-expressed targets, the SAC-IS related protein-protein interaction network (PPI) was constructed and the main biological functions and signal pathways of SAC were explored. Then hub genes were extracted by cytoHubba plug-in in Cytoscape 3.7.1 and used for molecular docking through AutoDock 4.2.6. Based on the results of molecular docking and signal pathways analysis, potential targets were further verified by the experiment.

## Materials and Methods

### Collection of the Pharmacokinetic Information by TCMSP

TCMSP (http://tcmspw.com/tcmsp.php) is a powerful knowledge repository of systems pharmacology for TCM, which provide information about compounds, ADME-related (absorption, distribution, metabolism, and excretion) properties, targets and diseases of Chinese herbal medicine with potential biological effects (Ru et al., 2014). We used “salvianolic acid C” as the keyword to search its pharmacological and molecular properties by TCMSP.

### Prediction of SAC-Related Targets

SwissTargetPrediction (http://www.swisstargetprediction.ch/) is a database which can accurately predict the targets of bioactive molecules based on a combination of 2D and 3D similarity measures with known ligands (Gfeller et al., 2014). Through comparing the molecular similarity with the active compounds of SAC, the SAC-related targets were selected. Meanwhile, PharmMapper can obtain potential targets and predict the biological activity of compounds by using active small molecules as probs (Wang et al., 2017).

PubChem (https://pubchem.ncbi.nlm.nih.gov/) is a public repository for biological activity data of small molecules (Kim, 2016). Relevant files of SAC were downloaded from PubChem database and uploaded to SwissTargetPrediction and PharmMapper databases respectively. The common potential drug targets predicted by the two databases were selected for further verification.

### Prediction of IS-Related Targets

DisGeNET (https://www.disgenet.org/) (Piñero et al., 2017), DrugBank (https://www.drugbank.ca/) (Wishart et al., 2018), OMIM (http://www.omim.org/) (Amberger et al., 2019), and TTD (http://db.idrblab.net/ttd/) (Wang et al., 2020) were all selected to obtain IS-related targets. These databases contain rich and cutting-edge disease-related targets. All databases were searched with the keyword “ischemic stroke”, and the common genes related to IS were selected as candidate targets.

### PPI Network Construction

STRING (https://string-db.org/) (Szklarczyk et al., 2017) aims to collect and integrate the information of all functional interactions between the expressed proteins. The SAC-related targets and IS-related targets obtained in the above steps were analyzed and compared. Then the overlapping genes of targets were extracted and imported into STRING 11.0, and “Homo sapiens” was selected from optional species with minimum interaction score as medium confidence (0.4). Then drug-disease target PPI network was obtained.

### Gene Function and Pathway Enrichment Analysis

The list of co-expressed target genes was input into DAVID Bioinformatics Resources 6.8 (https://david.ncifcrf.gov/) (Huang et al., 2007) for Gene ontology (GO) with Q value <0.01 and Kyoto Encyclopedia of Genes and Genomes (KEGG) enrichment analysis with Q value <0.05.

### Construction of Networks

Through the information obtained in the above steps, Cytoscape 3.7.1 software (Shannon et al., 2003) was used to construct the association between active compound (SAC), candidate targets and related KEGG pathways (SAC-target-pathway network), so that we could intuitively understand the interaction relationship of each node. Furthermore, the top 20 hub genes were predicted using the cytoHubba plug-in in Cytoscape 3.7.1.

### Molecular Docking

Molecular docking is a computer-aided drug design method (Li T. et al., 2021). It can simulate the interaction between small molecule ligands and biological macromolecular receptors, and predict the binding mode and affinity between them (Udrea et al., 2021a; Udrea et al., 2021b), so as to screen the lead drugs that can bind to the target, and provide guidance for reasonably optimizing the molecular structure of drugs, which is of great significance for drug research and development.

The crystal structures of top 20 hub genes were obtained from RCSB Protein Data Bank (http://www.rcsb.org/), and the 3D structures of SAC was retrieved from PubChem database (https://pubchem.ncbi.nlm.nih.gov/). SAC was ligands, and the 20 hub genes were receptors. The receptors were introduced to PyMOL 2.4 (https://pymol.org/2/) and AutoDock 4.2.6 (http://autodock.scripps.edu/) (Bitencourt-Ferreira et al., 2019) for removal of water molecules and heteroatoms, and addition of charges and hydrogen atoms. Subsequently, the conformations of ligand-receptor binding were predicted by AutoDockTools. Binding energy, one of the results of molecular docking, was used to evaluate the potential of ligand-receptor binding (usually ≤ -5 kcal/mol). The conformation with the best binding energy was chosen as the final conformation and visualized with PyMOL 2.4.

### Animal

Adult male Sprague Dawley rats (body weight, 300 ± 20 g) were obtained from Shanghai Sippr-BK Laboratory Animal Co., Ltd. (Shanghai, China). All animals were raised on a 12/12 h light/dark cycle with a room temperature of 25 ± 1°C and humidity of 60–65%, and allowed free access to food and water. The above were strictly in accordance with the National Institutes of Health (NIH) Guide for the Care and Use of Laboratory Animals (Guide for the Care and Use of Laboratory Animals, 2011). The approved protocols and guidelines of animal experiments were obtained from the Institutional Animal Care and Use Committee of the Laboratory Animal Research Center of Zhejiang Chinese Medical University.

### Materials

SAC (purity ≥98%, CAS No. 115841–09–3) were purchased from Nanjing Shizhou Biotechnology Co., Ltd (Nanjing, China), and 2, 3, 5-triphenyl tetrazoliumchloride (TTC) (Lot No. BCCB1241) was from Jiancheng Biotech Co. (Nanjing, China). Primary antibodies against VEGFR2 (gene name: KDR) (Cat No: 2479), PPARγ (gene name: PPARG) (Cat No: 2435), MMP1 (Cat No: 54376) and secondly antibodies (Cat No: 7074) were obtained from Cell Signaling Technology (Danvers, MA, United States), IGF1 (ab134140) was from Abcam (Cambridge, MA, United States), and actin (C-2) (sc-8432) was from Santa Cruz Biotechnology, Inc (Dallas, TX, United States).

### Establishment and Administration of Cerebral Ischemia/Reperfusion (I/R) Injury

Middle cerebral artery occlusion (MCAO) was established by Longa’s method (Longa et al., 1989) to induce cerebral I/R injury. In brief, rats were anesthetized with pentobarbital sodium (35 mg/kg, i. p.) after weighing, and placed on a heating pad with the body temperature maintained at 37 ± 0.5°C. After the skin incision, the right common carotid artery (CCA), external carotid artery (ECA) and internal carotid artery (ICA) were exposed. Then we ligated the CCA and ECA, and gently inserted a nylon monofilament with rounded tip (diameter of 0.28 mm, Beijing Cinontech Biotech Co., Ltd., Beijing, China) through the incision in the CCA into ICA until the middle cerebral artery (18–20 mm). And the nylon monofilament was removed to allow reperfusion after 1 hour of MCAO. The following steps of surgery were to suture the incision, and maintain the body temperature of rats until they woke up.

The rats were randomly divided into sham operation group (sham group), I/R group and I/R injury with SAC treatment group (SAC group), with six rats in each group (3 rats for TTC staining, and the other three for western blot). 20 mg/kg of SAC was administered intraperitoneally to the SAC group for the next 7 days. Sham group and I/R group were given equal amount of normal saline in the same way.

### Neurological Deficit Score and TTC Staining

After 7 days of administration, the neurological deficit score of rats was scored according to Longa’s method (0 = no neurologic deficit; 1 = failure to extend the left forepaw; 2 = rats circled to the left when crawling; 3 = rats leaned to the left when standing; 4 = rats were unable to walk spontaneously, and lost consciousness) (Longa et al., 1989). Then rats in each group were deep anesthetized by pentobarbital sodium (35 mg/kg, i. p.), of which brains were removed and stored at a temperature of −20°C for 15 min. The brains were cut into six pieces with a thickness of about 2 mm, then placed in 2% TTC at 37°C for 30 min. The staining results were photographed and analyzed by ImageJ software (https://imagej.nih.gov/ij/). The brain infarct volume rate was calculated by measuring the percentage of infarct volume in the total volume of slices.

### Western Blot

According to the molecular docking and KEGG analysis results, we selected the four core target genes with strong binding energy to SAC for Western blot verification. The right brain tissue of rats was added with ice-cold lysis buffer containing protease inhibitor, fully ground and centrifuged (12,000 rpm, 4°C, 20 min). The protein concentration was determined by BCA protein assay kit (Thermo Fisher Scientific Inc., Shanghai, China). After denaturation by boiling, the proteins were separated by sodium dodecyl sulfate-polyacrylamide gel electrophoresis (SDS-PAGE) and transferred to 0.45 μm polyvinylidene fluoride (PVDF) membranes (Immobilon-P, Merck Millipore Ltd.). Then membranes were blocked with 10% skimmed milk at room temperature for 1 h and then incubated with anti-VEGFR2 (1:1,000 dilution), anti-IGF1 (1:1,000 dilution), anti-PPARγ (1:1,000 dilution), anti-MMP1 (1:1,000 dilution), and actin (C-2) (1:1,000 dilution) overnight at 4°C. Subsequently, the membranes were incubated with horseradish peroxidase-linked secondary antibody (1:5,000 dilution) at room temperature for 1 h. The results of Western blot were visualized by Azure 300 Gel Imaging System (Azure Biosystems Inc., United States) and analyzed by ImageJ software.

### Statistical Analysis

The results were expressed as mean ± standard deviation (SD) and analyzed by one-way analysis of variance (ANOVA) followed by Tukey’s post hoc test or Kruskal-Wallis test with SPSS 25.0 software (SPSS Inc., United States). A value of *p* < 0.05 was considered to be statistically significant.

## Results

### Pharmacokinetic Information of SAC

The pharmacological and molecular properties of SAC were acquired using TCMSP (Table 1), including molecular weight (MW), low lipid/water partition coefficient (AlogP), hydrogen bond donors (Hdon), hydrogen bond acceptors (Hacc), oral bioavailability (OB), Caco-2 permeability (Caco-2), blood brain barrier (BBB), and drug likeness (DL). Generally, according to the parameter information and screening criteria of TCMSP database (Ru et al., 2014; Li et al., 2018), the DL of effective drugs should be greater than 0.18. Obviously, SAC showed good drug-likeness and deserves for further research.

**TABLE 1:** Pharmacological and molecular properties of SAC.

Name	MW	AlogP	Hdon	Hacc	OB (%)	Caco-2 (nm/s)	BBB	DL
SAC	492.46	4.53	6	10	2.50	−0.23	−1.02	0.81

### Candidate Targets

**FIGURE 2:**
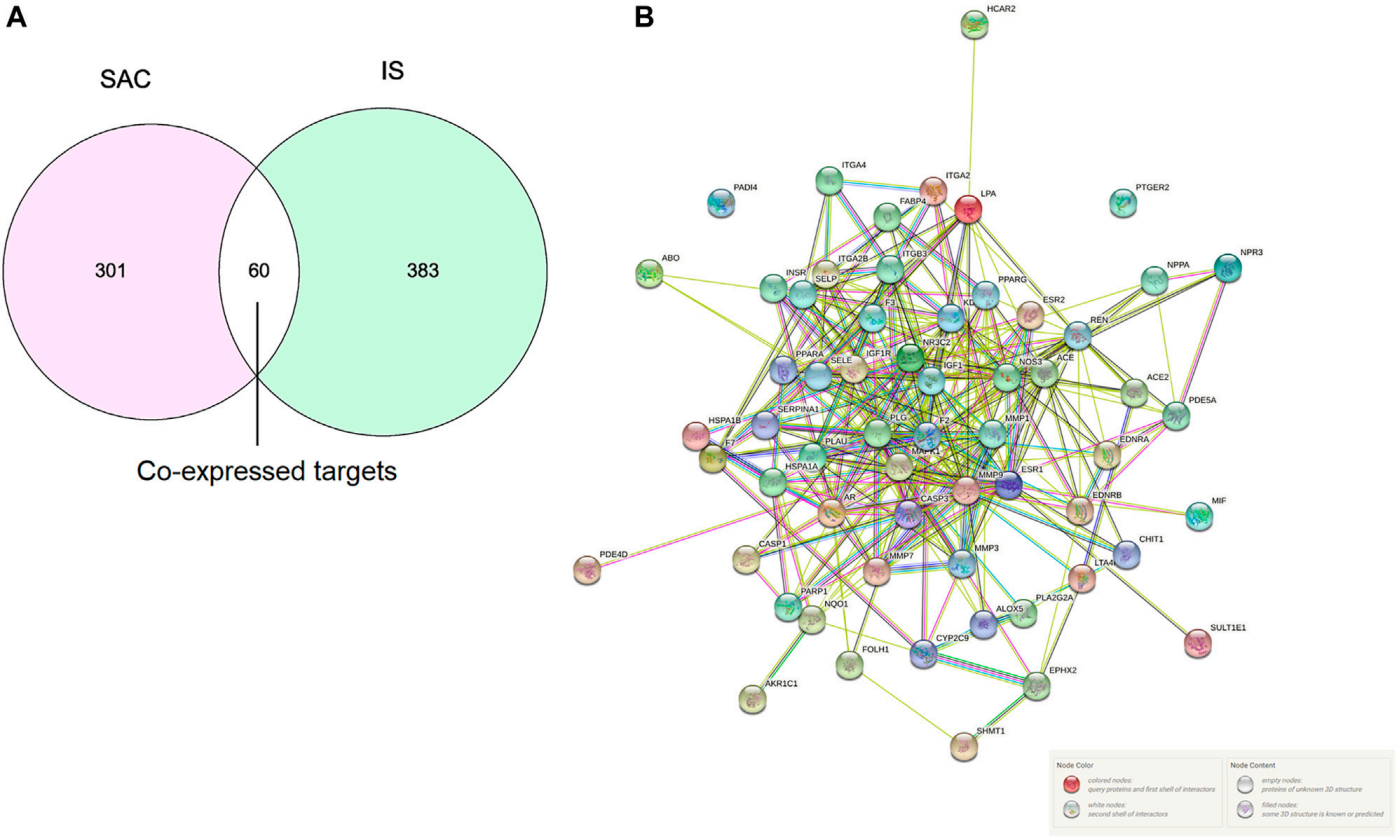
Intersection targets of SAC and IS **(A)**. PPI network, Nodes represent proteins, and edges represent interactions **(B)**.

A total of 361 potential SAC-related targets were predicted for further verification by SwissTargetPrediction and PharmMapper, and a total of 443 IS-related targets were obtained from DisGeNET, DrugBank, OMIM, and TTD databases. 60 overlapping genes of targets were identified as co-expressed targets (Figure 2A).

### PPI Network Analysis

The PPI network analyzed by STRING 11.0 was shown in Figure 2B. The proteins in PPI network are represented by circular nodes with protein 3D structure inside, and the lines between nodes represent the interaction between proteins. The more lines, the stronger the interaction between the two. A total of 60 genes were input, of which 58 nodes interacted, and the other two nodes (PADI4 and PTGER2) were not associated with other nodes.

### GO and KEGG Pathway Enrichment Analysis

**FIGURE 3:**
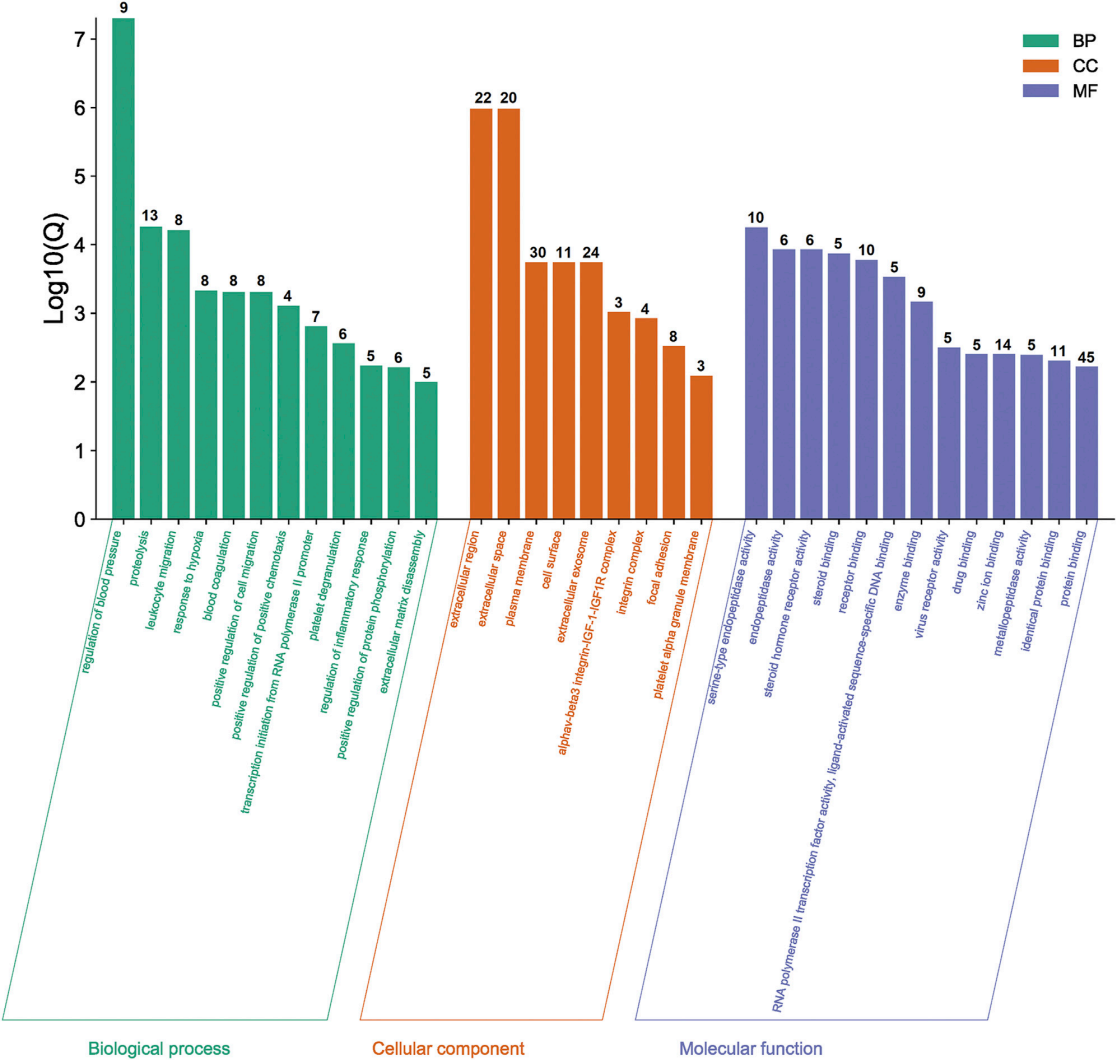
Results of GO enrichment analysis. The number of genes in each category is shown on top of each bar.

The functional and pathway enrichment information for the potential genes were analyzed by DAVID Bioinformatics Resources 6.8. The results of GO enrichment analysis were shown in Figure 3. Taking Q value <0.01 as the screening condition, we finally obtained 34 GO terms, including 12 biological processes, nine cellular components, and 13 molecular functions. Moreover, these terms mainly focused on proteolysis, plasma membrane, protein binding, etc.

**FIGURE 4:**
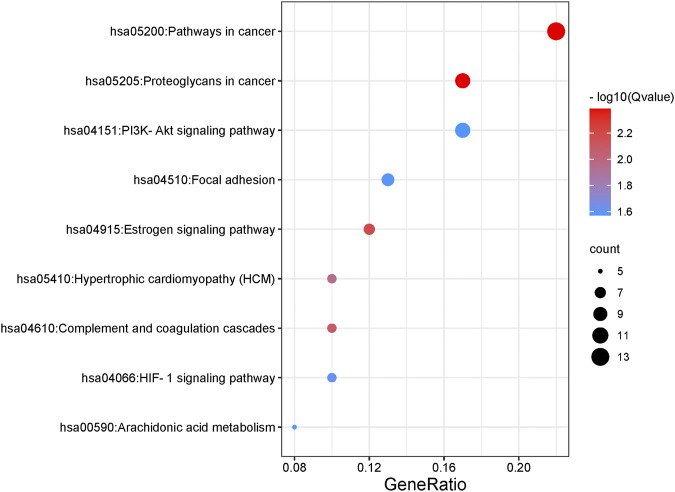
Results of top nine KEGG pathway enrichment.

As for KEGG pathways analysis, top nine related signaling pathways (Q value <0.05) were listed in Table 2 and Figure 4, and mainly concentrated in proteoglycans in cancer, pathways in cancer, PI3K-Akt signaling pathway, Focal adhesion, etc.

**TABLE 2:** The enriched KEGG pathway and the related genes.

Pathway	Genes	Fold enrichment	Q value
Proteoglycans in cancer	PLAU, CASP3, ITGA2, ITGB3, KDR, MAPK1, IGF1, ESR1, MMP9, IGF1R	6.03	0.0041
Pathways in cancer	MMP1, PTGER2, ITGA2, ITGA2B, IGF1, MMP9, IGF1R, AR, EDNRA, EDNRB, CASP3, MAPK1, PPARG	3.99	0.0041
Estrogen signaling pathway	NOS3, MAPK1, ESR1, MMP9, ESR2, HSPA1B, HSPA1A	8.53	0.0066
Complement and coagulation cascades	F7, SERPINA1, PLAU, PLG, F2, F3	10.49	0.0081
Hypertrophic cardiomyopathy (HCM)	ACE, ITGA4, ITGA2, ITGB3, ITGA2B, IGF1	9.28	0.0115
HIF-1 signaling pathway	NOS3, INSR, NPPA, MAPK1, IGF1, IGF1R	7.54	0.0247
Focal adhesion	ITGA4, ITGA2, ITGB3, ITGA2B, KDR, MAPK1, IGF1, IGF1R	4.69	0.0260
Arachidonic acid metabolism	CYP2C9, ALOX5, EPHX2, PLA2G2A, LTA4H	9.89	0.0260
PI3K-Akt signaling pathway	ITGA4, NOS3, ITGA2, ITGB3, INSR, ITGA2B, KDR, MAPK1, IGF1, IGF1R	3.50	0.0269

### SAC-Target-Pathway Network

**FIGURE 5:**
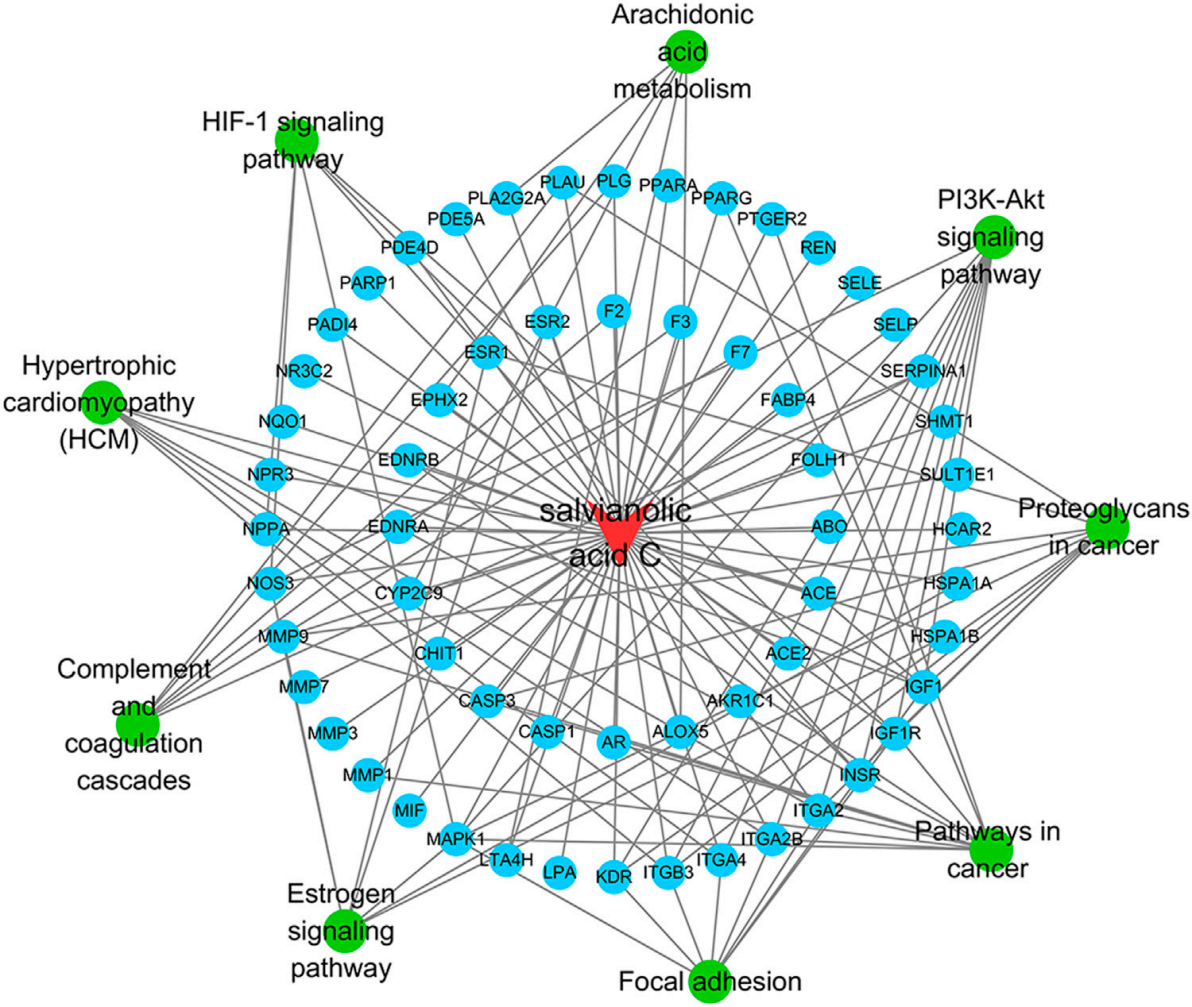
SAC-target-pathway network. The red V shape, green circles, and blue circles represent SAC, targets, and KEGG pathway, respectively.

**FIGURE 6:**
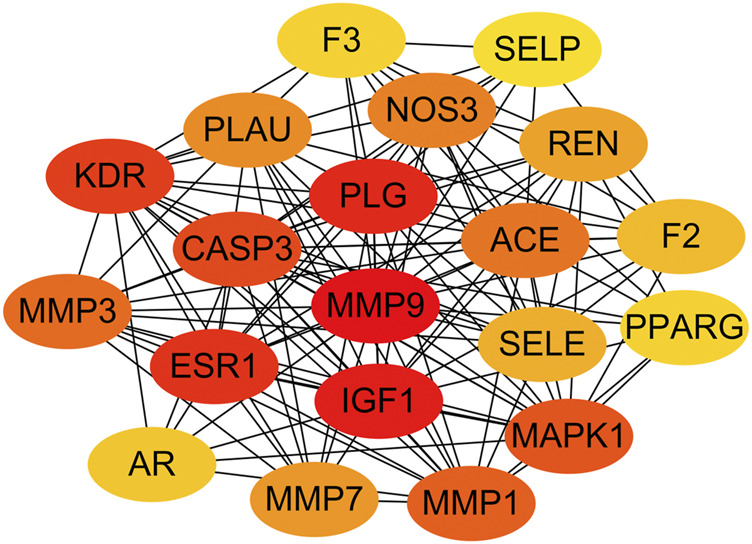
Top 20 hub genes.

The SAC-target-pathway network contained a total of 70 nodes (Figure 5). Nodes with different colors and shapes represent different active compound, targets and pathways, including one compound (red V shape) and 60 targets (green circle) and nine KEGG pathways (blue circle). And the number of lines between nodes represent the importance of nodes in the network. Meanwhile, top 20 hub genes were calculated using the cytoHubba plug-in in Cytoscape 3.7.1 and visualized in Figure 6. As shown, the node color represented the score of nodes, the darker and redder nodes meant a greater score.

### Molecular Docking Results

**FIGURE 7:**
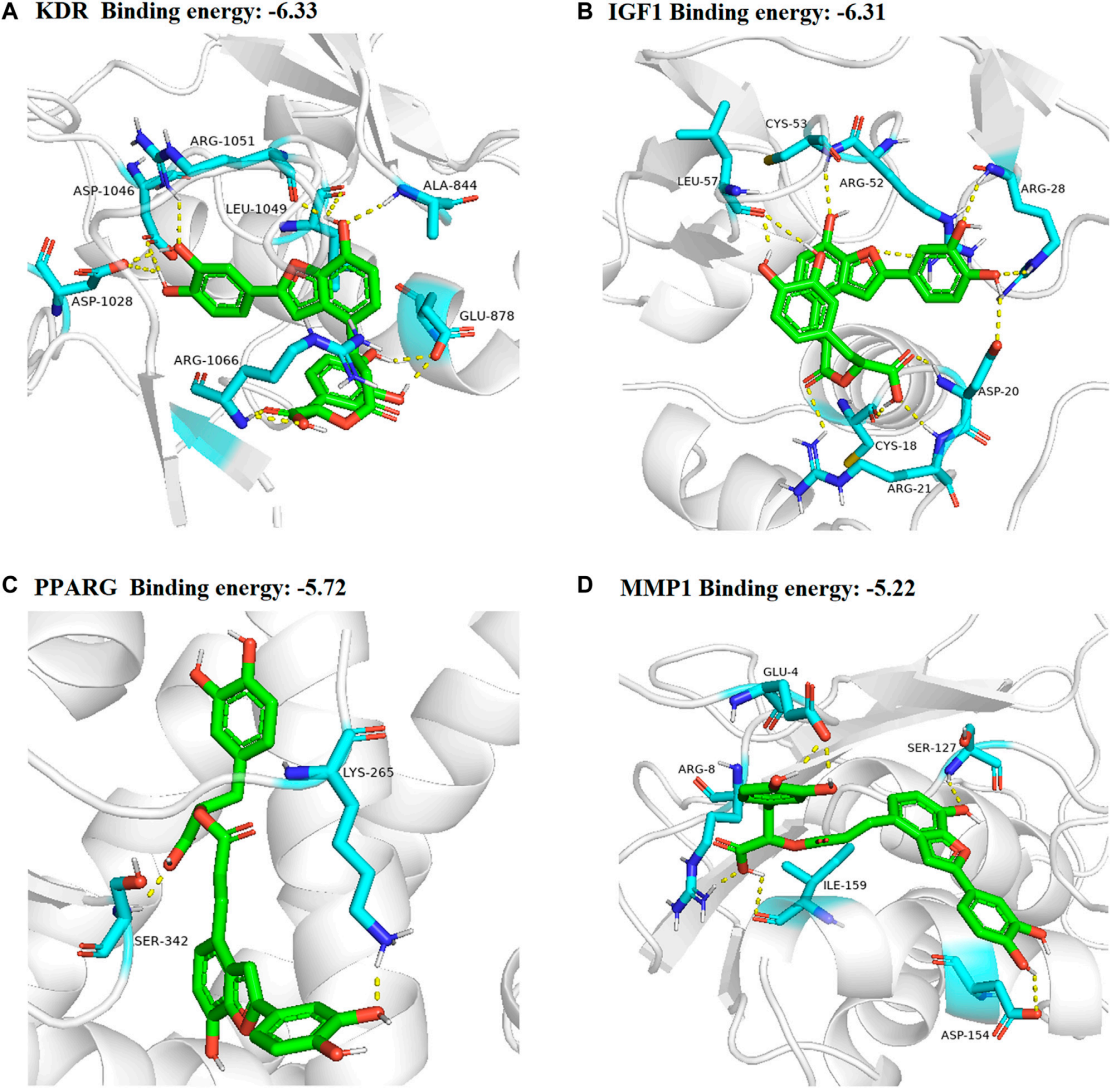
3D docking conformation of SAC with **(A)** KDR, **(B)** IGF1 **(C)** PPARG **(D)** MMP1, respectively. The green sticks represent SAC.

In total, top 20 hub genes selected (Figure 6) and SAC were carried out using AutoDock 4.2.6. for molecular docking. The binding energies between target proteins and SAC were in the range of −7.18 – −2.09 kcal/mol. According to the screening conditions for docking results (He et al., 2020; Wang et al., 2021), only nine target proteins had binding energy less than - 5 kcal/mol with SAC (Table 3). Eventually, compared with the important targets enriched by KEGG pathway, four targets (KDR, IGF1, PPARG, and MMP1) were selected and displayed in a 3D graph (Figure 7). There were multiple hydrogen bonds (the yellow dotted lines represent hydrogen bonds) between the core target proteins and SAC. As shown, SAC was bound to ALA-844, ARG-1051, ARG-1066, ASP-1028, ASP-1046, GLU-878, and LEU-1049 binding sites of KDR, ARG-21, ARG-28, ARG-52, ASP-20, CYS-18, CYS-53, and LEU-57 binding sites of IGF1, LYS-265 and SER-342 binding sites of PPARG, ARG-8, ASP-154, GLU-4, ILE-159, and SER-127 binding sites of MMP1.

**TABLE 3:** The docking information of nine targets with SAC.

Gene	Binding energy	Gene	Binding energy
F2	−7.18	PPARG	−5.72
MMP7	−6.40	PLG	−5.60
KDR	−6.33	ACE	−5.42
IGF1	−6.31	MMP1	−5.22
REN	−5.82	—	—

### Assessment of I/R Injury Model

**FIGURE 8:**
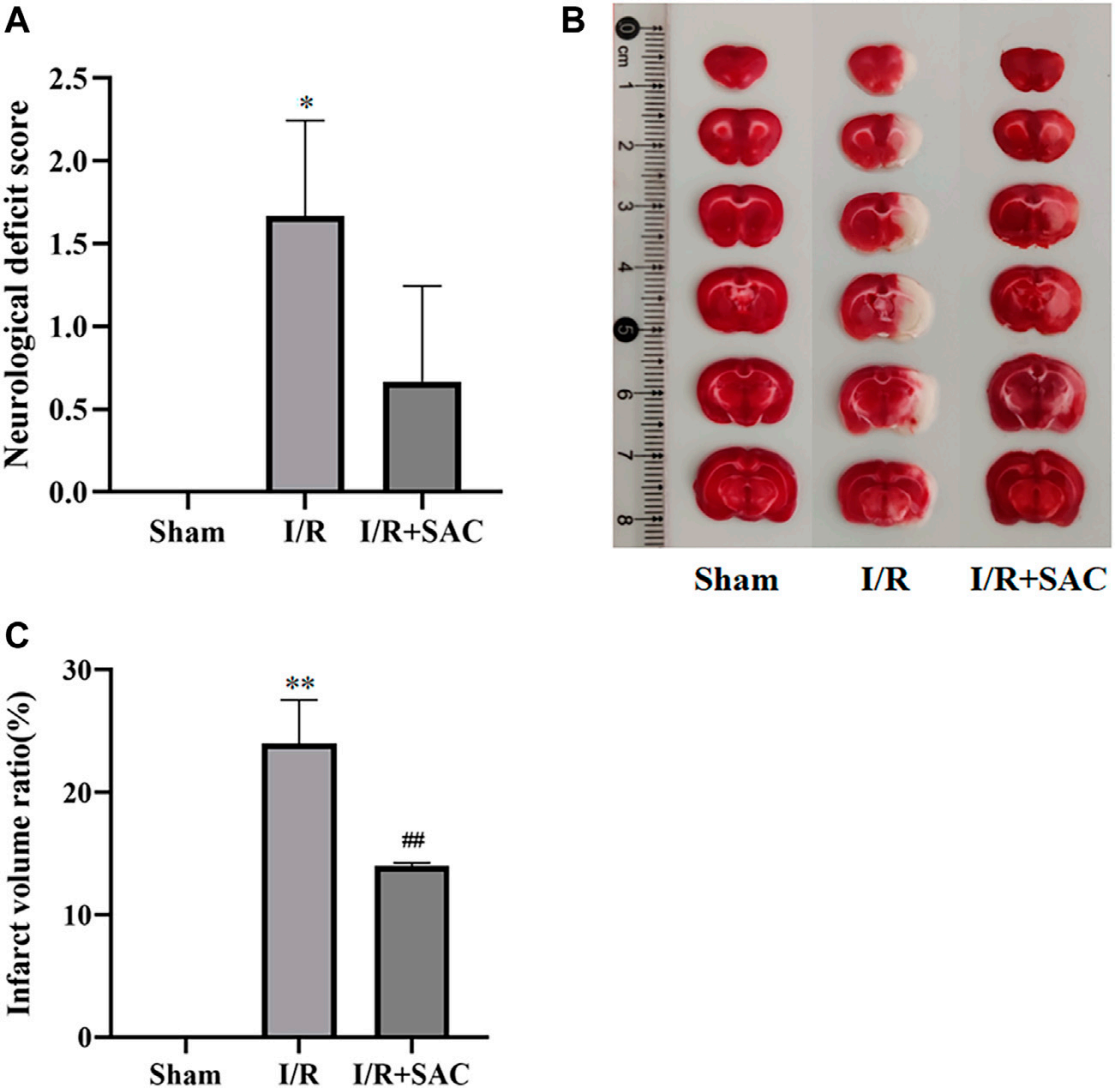
Neurological deficit score of rats in each group after 7 days of SAC treatment **(A)**. Representative photographs **(B)** and statistical results **(C)** of brain tissue in rats. *p < 0.05, **p < 0.01, vs the sham group. ^#^ *p* < 0.05, ^##^ *p* < 0.01, vs the I/R group (n = 3).

As shown in Figure 8A, the neurological deficit score of the I/R group was significantly higher than that of the sham group, and there was significant difference between the two groups (*p* < 0.05). After 7 days of SAC treatment, the neurological deficit score in the SAC group was significantly lower than that in the I/R group, but there was no significant difference.

As shown in Figure 8B, the normal tissue of brain was deep red by TTC staining, while the infarcted area was white. Compared with the sham group, the infarct volume in the I/R group increased significantly (*p* < 0.01); Moreover, the infarct volume in the SAC administration group was significantly reduced compared with that in the I/R group (*p* < 0.01) (Figure 8C).

### Expression of Core Target Proteins

**FIGURE 9:**
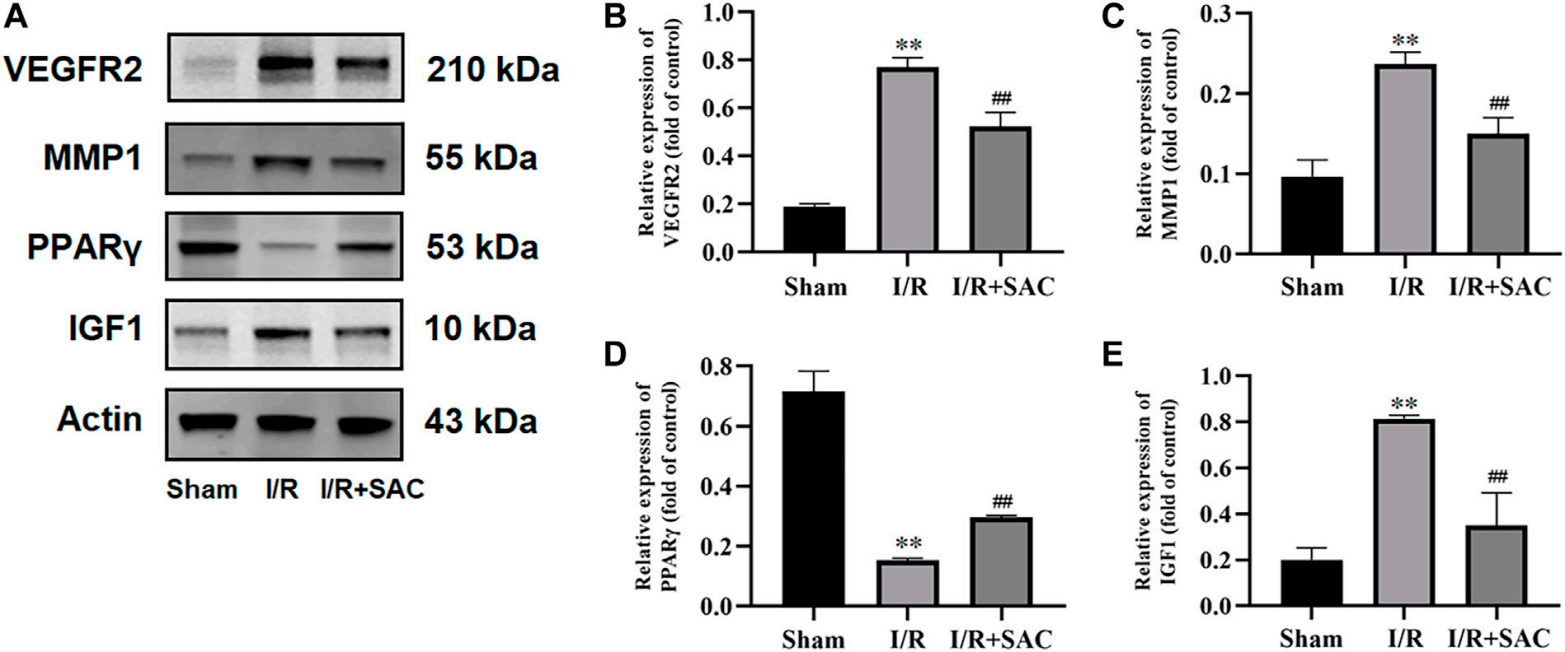
Representative images from western blot for the proteins **(A)** and relative expression levels of VEGFR2 **(B)**, MMP1 **(C)**, PPARγ **(D)**, and IGF1 **(E)**. **p < 0.01, vs the sham group. ^#^ *p* < 0.05, ^##^ *p* < 0.01, vs the I/R group (n = 3).

As shown, the expression level of VEGFR2, MMP1 and IGF1 (Figures 9A–C, E) in the I/R group were increased significantly when compared with the sham group (*p* < 0.01), while the SAC group decreased significantly compared with the I/R group (*p* < 0.01). Moreover, compared with the sham group, the protein expression level of PPARγ (Figures 9A, D) were markedly down-regulated in the I/R group (*p* < 0.01), while the SAC group markedly up-regulated compared with the I/R group (*p* < 0.01).

## Discussion

In recent years, network pharmacology has become an important method to study the relationship between diseases and drugs and drug discovery (Tao et al., 2020; Zhou et al., 2020). In this study, network pharmacology and molecular docking methods were used to screen the molecular mechanism of SAC against IS. Firstly, it can be seen from the ADME-related properties of SAC that its oral bioavailability is low. Therefore, we chose intraperitoneal injection in animal experiments to promote the absorption of drugs in rats. And the value of BBB greater than −0.3 is considered to be penetrating (Tattersall et al., 1975), so the BBB score of SAC is poor. While its DL is very high, and we have indeed proved through pharmacological experiments that it could improve the condition of cerebral infarction and neurological function. So, its efficacy may not be a direct effect, which requires further experimental exploration. Secondly, by combining multiple drug databases and disease databases, we preliminarily obtained 60 overlapping targets (Figure 2A) for the construction of PPI network, we found 58 nodes had protein-protein interaction (Figure 2B). And KEGG analysis revealed that pathways of SAC against IS include Proteoglycans in cancer, Pathways in cancer, Estrogen signaling pathway, Complement and coagulation cascades, Hypertrophic cardiomyopathy (HCM), HIF-1 signaling pathway, Focal adhesion, Arachidonic acid metabolism, PI3K-Akt signaling pathway, etc. It can be seen that these pathways are mainly related to anti-inflammatory, anti-apoptosis, anti-oxidant stress (Duronio, 2008; Huang et al., 2018; Liu et al., 2019) and other physiological and pathological processes, which are also closely related to the pathological process of IS (Zhou et al., 2021). Then 20 hub genes screened by cytoHubba plug-ins were used for molecular docking, the docking results indicated that SAC may effectively interact with the nine targets (F2, MMP7, KDR, IGF1, REN, PPARG, PLG, ACE and MMP1). Consequently, we compared the results molecular docking with KEGG analysis and selected four important genes for further verification.

As for the four core targets, existing reports have confirmed that they may be closely related to cerebral I/R injury and recovery. For example, vascular endothelial growth factor receptor 2 (VEGFR2), also called as fetal liver kinase receptor 1 (Flk-1)/kinase domain receptor (KDR) (Geiseler and Morland, 2018), is a kind of receptor of vascular endothelial growth factor (VEGF). It can activate endothelial nitric oxide synthase (eNOS) pathway and reduce the expression of tight junction protein to induce vascular permeability, which is an important regulator of angiogenesis and vascular permeability (Li et al., 2011; Huang et al., 2020). In the pathological state of cerebral ischemia injury, VEGF and VEGFR2 are mainly overexpressed in endothelial cells, resulting in the destruction of tight junction, further leading to the destruction of BBB and vascular brain edema (Lafuente et al., 2006; Wang et al., 2019; Liu et al., 2020). Thus, our results showed that the protein expression of VEGFR2 in SAC treatment group was significantly lower than that in I/R group (Figures 9A, B), which is consistent with the previous experimental results (Wang et al., 2019; Liu et al., 2020) and suggested that SAC may inhibit the overexpression of VEGFR2 after cerebral I/R injury. Matrix metalloproteinase 1 (MMP1) is an important collagenase, which is highly active in extracellular matrix, vascular remodeling and angiogenesis (Mazor et al., 2013). Activated MMP1 can up-regulate the expression of VEGFR2, induce endothelial cell proliferation and promote the expression of angiogenesis genes (Mazor et al., 2013; Fan et al., 2020). It has been reported that the expression of MMP1 in patients with acute stroke is significantly increased (Kouwenhoven et al., 2001) and MMP1 polymorphism is a potential marker for predicting stroke (Huang et al., 2017). In addition, the previous experiment has shown that the increased expression of MMP1 leads to the development of brain injury and neurological deficit (Victoria et al., 2020). As shown in Figures 9A, C, the protein expression of MMP1 in I/R group was significantly higher than that in sham group, but the expression of MMP1 may be inhibited to a certain extent after SAC treatment. As for Peroxisome proliferator-activated receptor gamma (PPARγ) is a nuclear transcription factor widely expressed in macrophages and microglia (Li L. et al., 2021). Moreover, it is considered to be an effective therapeutic target for cerebral ischemia (Villapol, 2018; Gamdzyk et al., 2020; Li L. et al., 2021). In addition to its recognized anti-inflammatory effect, it can also reduce oxidative stress, BBB injury, apoptosis, and promote angiogenesis and neurogenesis (Ballesteros et al., 2014; Villapol, 2018; Gamdzyk et al., 2020). To explore whether SAC passes PPARγ-mediated pathway to promote the recovery of cerebral ischemic injury, some rats with MCAO were treated with SAC and then the protein expression of PPARγ was verified. The results showed that the protein expression level of PPARγ was markly up-regulated after cerebral I/R, which indicated that SAC may pass PPARγ-mediated pathway to play a neuroprotective role in cerebral I/R injury. Insulin-like growth factor-1 (IGF1) is an important growth factor, which can promote the differentiation, proliferation, myelination and neurite growth of neurons and oligodendrocytes, and reduce apoptosis (Bake et al., 2014; Cui et al., 2016). After focal cerebral ischemia injury, IGF1 plays an important role in promoting the proliferation of neural progenitor cells and its protein expression increased in the activated astrocytes (Yan et al., 2006). At the same time, it can activate PI3K-Akt signaling pathway and further mediate cell survival (Bake et al., 2014). Compared with the sham group, the protein expression of IGF1 in the I/R group was up-regulated, indicating that IGF1 was activated after cerebral ischemia; while the expression of IGF1 in SAC group decreased significantly when compared with I/R group, suggesting that SAC treatment may contribute to the recovery of cell function after stroke and the sensitivity of IGF1 decreased. Based on the Western blot verification results, we can speculate that mechanism of SAC in the treatment of IS aims to up-regulate the expression of PPARγ, and down-regulate the expression of VEGFR2, MMP1, and IGF1.

## Conclusion

Our study conducted a systematic and comprehensive study on the mechanism of SAC in the treatment of IS for the first time. The 20 hub genes and related biological function and nine major KEGG pathways of SAC against IS were studied by network pharmacology. At the same time, nine potential targets for effective binding to SAC were obtained by molecular docking. In experiment, the neurological function of rats treated with SAC was improved, and the volume of cerebral infarction was significantly reduced. Four target proteins (VEGFR2, MMP1, PPARγ and IGF1) after western blot verification also showed the role of SAC in anti-inflammatory and angiogenesis. All the results provided new insights for clarifying the molecular mechanism of SAC, and also provided a reference for the research and development of anti-IS drugs.

## Data Availability

The original contributions presented in the study are included in the article/supplementary materials, further inquiries can be directed to the corresponding authors.
